# Rabies Post-Exposure Prophylaxis in the Philippines: Health Status of Patients Having Received Purified Equine F(ab')_2_ Fragment Rabies Immunoglobulin (Favirab)

**DOI:** 10.1371/journal.pntd.0000243

**Published:** 2008-05-28

**Authors:** Beatriz P. Quiambao, Hazel Z. DyTioco, Ruby M. Dizon, Marilyn E. Crisostomo, Thelma M. Laot, Dirk E. Teuwen

**Affiliations:** 1 Research Institute for Tropical Medicine, Alabang, Manila, Philippines; 2 Sanofi Pasteur, Manila, Philippines; 3 College of Public Health, University of the Philippines, Quezon City, Manila, Philippines; 4 Sanofi Pasteur, Lyon, France; WHO-IVR, Switzerland

## Abstract

**Background:**

Recommended treatment for severe rabies exposure in unvaccinated individuals includes wound cleaning, administration of rabies immunoglobulins (RIG), and rabies vaccination. We conducted a survey of rabies treatment outcomes in the Philippines.

**Methods:**

This was a case series involving 7,660 patients (4 months to 98 years of age) given purified equine RIG (pERIG) at the Research Institute for Tropical Medicine (Muntinlupa, Philippines) from July 2003 to August 2004 following Category II or III exposures. Data on local and systemic adverse reactions (AR) within 28 days and biting animal status were recorded; outcome data were obtained by telephone or home visit 6–29 months post-exposure.

**Results:**

Follow-up data were collected for 6,464 patients. Of 151 patients with laboratory-confirmed rabies exposure, 143 were in good health 6–48 months later, seven could not be contacted, and one 4-year-old girl died. Of 16 deaths in total, 14 were unrelated to rabies exposure or treatment. Two deaths were considered PEP failures: the 4-year old girl, who had multiple deep lacerated wounds from a rabid dog of the nape, neck, and shoulders requiring suturing on the day of exposure, and an 8-year-old boy who only received rabies PEP on the day of exposure.

**Conclusions:**

This extensive review of outcomes in persons with Category III exposure shows the recommended treatment schedule at RITM using pERIG is well tolerated, while survival of 143 laboratory-confirmed rabies exposures confirms the intervention efficacy. Two PEP intervention failures demonstrate that sustained education and training is essential in rabies management.

## Author Summary

Infection from a bite by a rabid animal is fatal unless rapid treatment (thorough cleaning of the wound, administration of rabies immunoglobulins (RIG), and a full anti-rabies vaccination course) is provided. Ideally human RIG should be used, but cheaper, more readily available purified horse RIG (pERIG) are widely used in developing countries. Follow-up of over 7,600 patients previously given pERIG at the rabies treatment reference center in Manila (Philippines) provided updated health status for 6,458 patients 39 days to 29 months after treatment. A total of 151 patients had been bitten by animals with laboratory-confirmed rabies. Two rabies deaths were reported, one in a 4-year-old girl with bites on the back, shoulder, and neck so severe that stitching was required to prevent bleeding (against recommended practice), and another in an 8-year-old boy who only received rabies vaccination on the day of initial treatment. A 7-year-old cousin of this boy, bitten by the same animal, who did receive the full vaccination course was still healthy 10 months later. Fourteen other reported deaths had causes unrelated to rabies. These data illustrate the effectiveness of pERIG as part of the recommended treatment regimen, while highlighting the importance of adhering to current recommendations.

## Introduction

Rabies is a zoonotic disease characterized by progressive and incurable viral encephalitis, invariably fatal if untreated and usually transmitted by the bite(s) or scratches of an infected animal. Data from the Department of Health show that every year, over 100,000 people at risk in the Philippines receive rabies post-exposure prophylaxis (PEP), which varies according to the categorization of the exposure as defined by the World Health Organization (Table 1). The most severe cases, Category III, require wound cleaning, rabies vaccination, and direct wound infiltration with rabies immunoglobulin (RIG) and where possible, observation of the biting animal if it does not already display clinical symptoms of rabies for a period of 10 days [1],[2].

**Table 1:** WHO recommendations for suspected rabies post-exposure treatment [2].

Category	Type Of Contact With A Wild Or Domestic Animal Presumed To Be Rabid Or With Confirmed Rabies, Or An Animal Which Cannot Be Placed Under Observation	Recommended Treatment
I	Touching or feeding of animals; or licks on intact skin	None, if reliable case history is available
II	Nibbling of uncovered skin; or minor scratches or abrasions without bleeding	Administer vaccine immediately Stop treatment if animal remains healthy throughout an observation period of 10 days* or if animal is proven to be negative for rabies by a reliable laboratory using appropriate diagnostic techniques
III	Single or multiple transdermal bites; or Scratches and licks on broken skin Contamination of mucous membrane with saliva (*i.e.*, licks) Exposures to bats	Administer rabies immunoglobulin and vaccine immediately. Stop treatment if animal remains healthy throughout an observation period of 10 days or if animal is found to be negative for rabies by a reliable laboratory using appropriate diagnostic techniques

*: In Philippines, the recommended duration of the observation period is 14 days.

Infiltration of RIGs into the wound(s) is essential in the management of severe bites to provide passive antibody protection during the first 1–2 weeks while the body develops its own immune response to vaccination. The WHO recommends the use of human RIG (HRIG) or equine (ERIG) in category III exposures [2]. For multiple severe Category III exposure HRIG is recommended, however, when not available or accessible, ERIG or pERIG must be used. As availability of HRIG is constrained by the limited production capacities imposed when using human plasma as the immunoglobulin source, bite victims in highly endemic countries are more likely to receive ERIG or pERIG.

F(ab')_2_ fragment rabies immunoglobulin (Favirab, Sanofi Pasteur, Lyon, France) is a highly purified pERIG, characterized in animal models [3] and in humans [4] and is currently used in over 40 countries. Industrial chromatographic purification results in a product with a high purity with selective extraction of active immunoglobulin molecules (IgG) from plasma and a final purification of F(ab')_2_ from the IgG peptic digest. The final pasteurized solution for wound infiltration has a high specific activity, containing mainly F(ab')_2_ molecules (85%). The clearance of Favirab is more rapid than ERIG and HRIG, documented by certain experimental animal data, however, this is not considered to influence the efficacy. Fewer than 1% of patients report adverse events to Favirab, these consisting mainly of mild allergic type reactions.

We report the results of a review of consultation records and follow-up investigations to determine the health status of persons who received PEP, including Favirab as a source of ERIG, at the Research Institute of Tropical Medicine, Manila.

## Materials and Methods

For the purpose of this case series, only records of patients given commercial lots of pERIG (Favirab) at the Research Institute of Tropical Medicine (RITM) rabies Admitting Section from July 2003 to August 2004 were analyzed. RITM is a government research institution that serves as a major referral center for rabies and animal bite patients. The study was approved by the RITM Institutional Review Board. A verbal consent was obtained from the patient or parents/guardians of children immediately prior to the follow-up interview.

Patient records were retrieved from the Medical Records Department and reviewed by trained research assistants (under supervision of BQ). It was anticipated that some records could not be interpreted because of incomplete information contained in the patient's records, record loss or, simply, illegible handwriting. Data were transcribed on a standard data collection form developed for the study and included information on demographics, rabies exposure, animal status and/or laboratory investigation of the animal, rabies PEP including skin testing data, timing of pERIG and rabies vaccine administration, tetanus prophylaxis and local and systemic adverse reactions (ARs) occurring up to 28 days from the administration of pERIG. ARs were categorized as either not related or possibly, probably or definitely related. Skin testing was performed by intradermal (ID) administration of 0.1 mL of 1∶10 solution of pERIG and read after 15 minutes. A repeat skin test was performed in the event a result was positive or doubtful.

The pERIG dose was calculated at a dose of 40 iu/kg, infiltrated around the wounds. At the time of the case series study, any remaining pERIG was injected intramuscularly in the buttocks. Beginning November 2004, the recommendation on the administration of the remaining volume of the pERIG was modified and remaining pERIG was injected intramuscularly on the anterior thigh. The recommended rabies vaccination regimen was day 0, 3, 7, 28 and 90. Three different rabies vaccines were used, 99.1% Verorab vaccine, 0.7% Rabipur vaccine and 0.2% Lyssavac vaccine.

To document post-treatment health status, patients or parents/guardians of children were contacted either by telephone or by a home visit at least six months from the time of the bite.

### Rabies investigations

Whenever possible, as part of standard treatment procedures, and in order to confirm the presence of rabies virus in biting animals that had died or were killed, a direct Fluorescent Antibody Test (dFAT) was performed at the rabies laboratory of the RITM following standard procedures [5].

## Results

A total of 7,660 records of subjects having received pERIG at the time of their potential rabies exposure during the period July 2003 to August 2004 could be retrieved and were reviewed for this study; 3,502 (45.7%) subjects came from the Metro Manila area, 3,382 (44.2%) subjects came from the four neighboring provinces, Bulacan (in the north) Rizal (in the East), Cavite and Laguna (in the south) and the remaining 776 (10.1%) from other provinces as far as Camarines Norte located 350 km south of Metro Manila. The most affected age group was the under -10's, in which almost twice as many boys as girls were treated, and overall 61.8% of the cases reported were in children less than 15 years of age. The length of follow-up varied between 35 days and 29 months.

### Review of data contained in the patient's records at RITM

#### pERIG administration

The results of skin testing prior to pERIG administration were documented for 7,495 subjects (97.8% of the study population). A positive result (erythema ≥6 mm) was found in five subjects (0.07%) and inconclusive results were recorded for three subjects, but all 8 subjects were negative upon repeat testing and no hypersensitivity reactions were noted following the administration of pERIG.

The route of administration of pERIG was documented in 7,639 subjects (99.7%); combined wound infiltration and intramuscular injection in 7,470 (97.5%) subjects, infiltration into the wounds alone in 108 (1.4%) subjects, and intramuscular injection only in 61 subjects (0.8%).

#### Rabies vaccination and tetanus prophylaxis

A total of 7,542 subjects (98.5%) received rabies vaccine on Day 0: 6,609 subjects (86.5%) by the intradermal (ID) route and 121 (1.6%) subjects by the intramuscular (IM) route, the route of administration not being documented in 796 (10.4%) records. The RITM records could only provide data on 4,118 (53.8%) subjects having received their second dose of vaccine on day 3, and 3,329 (43.5%) subjects having received their third dose on day 7. On day 28, 2,410 (31.5%) subjects returned for the 4^th^ dose and 687 (9.0%) subjects returned for the 5^th^ dose on day 90. It could not be ascertained whether 118 (1.5%) subjects with missing data received rabies vaccine elsewhere. These rabies vaccination data at RITM should be interpreted with caution and not regarded as a ‘lack of compliance’ or interpreted to suggest that fewer doses of vaccine are acceptable (or capable of conferring a high degree of protection), as patients frequently returned to their local community animal bite centers for follow-up vaccinations; hence information on subsequent doses would not have been entered into the RITM records.

Tetanus prophylaxis was provided to 6,342 (82.9%) subjects, as either tetanus toxoid or DTP vaccination, with administration of anti-tetanus serum in 5,652 (73.8%) subjects.

#### Safety

Of the 7,660 pERIG-treated subjects, local and systemic adverse reactions (AR) were documented in 35 (0.46%) and 104 (1.36%) subjects, respectively. Only 2 (0.03%) subjects had documented local ARs within 30 minutes post-vaccination and 11 (0.14%) subjects were documented as experiencing possible allergic reactions, such as dizziness, drowsiness, hypersensitivity reaction, itchiness, loss of consciousness and/or rash on the day of vaccinations. A total of 29 local and 90 systemic ARs were considered possibly, probably or definitely related to the pERIG and/or rabies vaccine administration. In addition, at the time of the survey, subjects reported on their ‘current’ medical condition. A total of 19 medical conditions were reported – nine general conditions, (*e.g.*, influenza, fever), three subjects with cancer, two subjects with nervous system disorders, *i.e.*, stroke and paralysis, two subjects with respiratory disorders, one liver disease, one kidney disease and one diabetes mellitus. When contact was attempted with the subject with paralysis in February 2007 to document his condition, we discovered that this 58-year-old man had died of complications of end-stage renal failure and diabetes mellitus.

#### Animal data

A total of 6,528 records (85.2%) reported data on the source of animal exposure. The most frequently identified animals associated with bites were dogs (73.2%), followed by cats (11.2%), other animals (0.54%), or combined dog and cat (0.05%). It is notable that 23 subjects (0.3%) received treatment after exposure to rabies-infected humans.

After a 14 day post-exposure observation period 3,922 (60.0%) animals were alive and considered normal; 1,561 (23.9%) animals died or were killed and the health status of the remaining animals could not be documented.

### Data obtained during the follow-up investigations

During follow-up investigations (by telephone, by home visit or by hospital record) a total of 7,604 (99.3%) subjects could be contacted as 56 subjects moved out of the area or their contact address or telephone number were inadequate. A total of 3,970 (51.8%) subjects were contacted by telephone, 3,595 (46.9%) subjects by home visit and 18 (0.23%) by telephone followed by a home visit.

Health status could be documented in 6,468 (84.8%) subjects, but for 1,164 (15.3%) subjects no follow-up information was obtained as they had moved from the original address, etc. The interval between PEP-and follow-up event varied between 35 days and 29 months.

#### Data on deaths

There were 16 deaths in the whole study population, two of which were considered rabies PEP intervention failures. The other 14 fatalities were considered not to be related to either rabies infection or PEP – seven due to cardiac conditions, two gastro-intestinal conditions, two cases of stroke, one case each of diabetes mellitus, tuberculosis, kidney failure and car accident. The time interval of the reported deaths varied between 35 days and 16 months (with a mean of 7.4 months).

**Figure 1:**
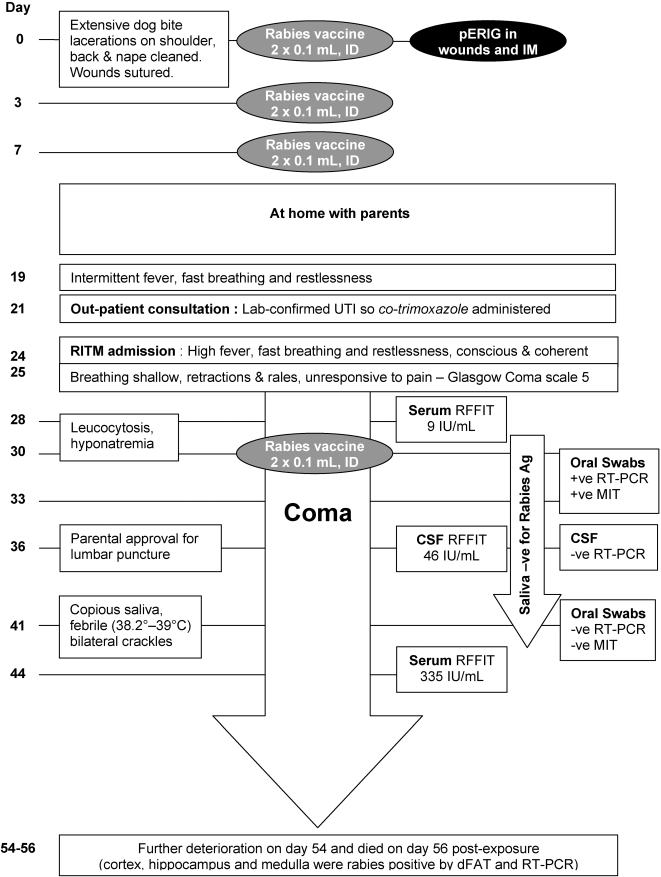
Clinical course of rabies fatality in a 4-year-old malnourished girl.

The clinical course of the first rabies fatality is summarized in Figure 1. Briefly, a 4-year-old malnourished girl was presented to the RITM 1½ hours after being attacked by a dog which had to be forcefully separated from the child by an uncle (who was also bitten). She had multiple deep lesions on the shoulder, back and nape. Immediate treatment consisted of wound washing, wound infiltration with diluted pERIG and intramuscular injection of the remaining volume, and rabies vaccine by ID route. Before returning home, the wounds were sutured because of their severity and continued bleeding. She received subsequent doses of rabies vaccine ID as scheduled. On day 24, however, she was hospitalized at RITM with signs and symptoms of rabies and died 55 days post-exposure.

On October 21^st^ 2003 the second case, an 8-year-old boy (from Laguna) was bitten by a pet dog and seen at RITM the same day with a Category III single laceration of the right eyelid. He received pERIG, partly infiltrated around the wound with the remaining volume administered IM in the buttocks (precise volume not recorded) and a first dose of rabies vaccine (0.1 mL ID in both deltoids). He also received anti-tetanus serum (3,000 IU IM), tetanus toxoid (0.5 mL IM) and cloxacillin (125 mg/mL, 11 mL every 6 hours). It was not documented whether wound cleaning was performed. A 7-year-old cousin bitten by the same dog resulting in a similar facial lesion received the same day 0 treatment. Both children returned with their parents to their home towns in Laguna. On November 20^th^ 2003, the 8-year-old boy presented with a moderate fever and was seen by a family practitioner. Five days later he was seen at the RITM Emergency Ward with restlessness, irritability and increased salivation. During consultation, hydrophobia and aerophobia could be elicited. The history revealed the boy had not received any further doses of rabies vaccine. The family refused hospital admittance; the boy died at home and was buried the same day, so no post-mortem investigations were conducted. No laboratory investigations on the animal had been initiated either. During follow-up contacts it was reported that the surviving 7-year-old cousin had completed the course of rabies vaccination until day 90 and was in good health 10.5 months later.

#### Data on laboratory confirmed rabies infection in animals

The remains of 252 animals, which together had inflicted wounds in 266 subjects, were tested for rabies virus infection by dFAT at RITM; 137 animals were rabies-positive; 96 were rabies-free and 19 were inconclusive.

#### Data on health status of subjects exposed to proven rabid animals

None of the 96 rabies-free animals inflicted wounds in more than one person, but attacks by the 137 rabies positive animals resulted in exposures of 151 subjects. The ages of these 151 individuals varied from 1 to 72 years with 68 (45%) being children less than 9 years of age. Whereas 149 (98.7%) of these subjects received pERIG by infiltration and IM administration, one subject received pERIG by infiltration alone and one received pERIG by by IM injection in the buttock since exposure consisted of kissing a rabid dog. A total of 147 (97.4%) subjects received a first dose of rabies vaccine, 82% by the ID route.

The health status was documented during the study survey period in 139 subjects with a mean observation period of 12.5 months (min 56 days–max 27 months). During the review process in February 2007, further attempts to identify and contact the remaining 12 subjects were made, including a review of national and regional rabies death registries, municipality death registries and discussions with officers of regional epidemiology surveillance units. We were then able to document survival and good health status in an additional five subjects, yielding a new total of 144 healthy subjects with a maximum observation period of 4 years. The remaining seven subjects could not be contacted; however, their names were not identified in the different death registries.

For comparison, in the group of 96 subjects bitten by laboratory-proven rabies-free animals, 86 persons were healthy and two subjects died; a 55-year-old man died of complications of diabetes 284 days post-exposure and a 37- year-old woman who died of a myocardial infarction 169 days post-exposure. Eight persons (8.3%) were lost to follow-up in this rabies-free group, a higher proportion than those lost to follow-up in the rabies-positive exposure group, (7/151, 4.6%) and only half of those lost to follow-up in the total population of the retrospective study (15.3%).

#### Location of the bites

The location and characteristics of the bites documented in subjects bitten by dFAT rabies-positive animals is summarized in Table 2, with over 54% of the bites in the richly innervated regions of head, hands or foot. Of the 138 subjects bitten in one body site, 68 (49%) suffered multiple wounds due to repeated biting by the rabid animal.

**Table 2:** Details of the body localization of the wounds inflicted by dFAT positive animals in one body site or at multiple sites in the body.

	Body site		# Total	%
	One	Multiple		
Head	10	5	15	9.9
**Upper Limb**	53	7	60	39.7
*arm*	*4*	*3*	*7*	*4.6*
*upper arm*	*2*	*0*	*2*	*1.3*
*lower arm*	*10*	*2*	*12*	*7.9*
*hand*	*37*	*2*	*39*	*25.8*
**Trunk**	9	0	9	6.0
*thorax*	*3*	*0*	*3*	*2.0*
*abdomen/penis*	*2*	*0*	*2*	*1.3*
*back, shoulder*	*3*	*0*	*3*	*2.0*
*buttock*	*1*	*0*	*1*	*0.7*
**Lower Limb**	66	1	67	44.4
*leg*	*21*	*1*	*22*	*14.6*
*thigh*	*10*	*0*	*10*	*6.6*
*lower leg*	*7*	*0*	*7*	*4.6*
*foot*	*28*	*0*	*28*	*18.5*
**Total**	**138**	**13**	**151**	**100**

## Discussion

This primary objective of the evaluation was to document the health status of subjects given rabies PEP which included use of the purified equine rabies immunoglobulin (pERIG), Favirab. The health status of patients treated after Category II/III exposure with the standard of care described in the RITM guidelines was documented by an active survey by telephone or home visits. The RITM guidelines are in accordance with the WHO guidelines [2],[6], the local Philippine recommendations and those developed in other countries [7].

Not unexpectedly, exposure was seen predominantly in children 5 years or younger, with over 23.28% of exposures occurring below 5 years of age; twice as many boys exposed when compared to girls. A similar age and sex distribution was reported in Thailand [8], although the age distribution was different to that recently reported in India [9].

The records made no reference to washing of the wounds, however, the standard recommendations of the RITM are likely to have been implemented carefully. Attending staff of the different animal bite treatment centers are trained in the appropriate management of patients with animal bites and those with Category III exposure are referred to the RITM Admitting Section. Wound washing is followed by an infiltration of pERIG into the wound site and injection of the remaining pERIG by IM route. This split administration was documented in 97.8% of the cases, only 108 cases (1.4%) having pERIG infiltration into the wound alone. 61 patients received pERIG by IM only; 16 of whom were exposed to rabid patients and 6 had healed wounds at the time of the consultation.

Over 54% of subjects had exposure in highly innervated body regions, such as the head, hands or feet, and one 23-year-old subject was bitten on the penis. Specific reference to exposure involving fingers or toes was documented in 24 subjects (2–61 years-old) and following treatment, no compartment syndrome was reported. This experience is similar to that observed in Thailand [10].

A first dose of rabies vaccine was administered to 98.5% of the study population. Rabies vaccination for the second and third dose was continued, as documented in the RITM records, in 53.8% and 43.5%, respectively. During the follow-up investigation, data on rabies vaccination in local animal bite treatment centers was not requested to avoid introducing a recall bias.

For the 6,468 subjects for whom follow-up information was obtained, the mean interval between exposure and follow-up was 11.5 months (ranging from 35 days to 29 months). During the survey window, 16 deaths were recorded in the whole study popualtion, in subjects from 4 to 72 years of age, occurring between 35 days and 16 months following exposure. The causes of 14 deaths were clearly identified as being unrelated to the rabies exposure, but two deaths were considered PEP intervention failures. Other clinical conditions reported by the patients or their parents occurred between 8 and 28 months but none were considered related to the treatment.

The documented healthy outcome of 143 subjects exposed to laboratory-proven rabid animals showed that the combination of pERIG, rabies vaccine and wound treatment is effective in protecting against canine rabies virus infection. The results confirm the clinical effectiveness of pERIG when used in PEP, notwithstanding the questions raised by scientific research, such as a more rapid clearance and a reported difference in protection associated with the use of pERIG against different rabies strains in animal models [11].

Nonetheless, no treatment intervention is 100% successful as illustrated by two tragic outcomes. The first case concerned a malnourished girl with severe lacerations in critical anatomical areas [12]. She was the first bite victim of the dog, and therefore probably received a large inoculum of the virus in highly innervated areas. Further, given her extensive lacerations with persistent bleeding, her wounds were sutured. She did subsequently develop an adequate immune response to the vaccine, as described for other malnourished children [13], but still succumbed to the rabies infection (Figure 1). The WHO recommendations (TRS931) state that in cases of multiple severe exposures, HRIG if available should be infiltrated in the wounds, otherwise pERIG should be used and a maximum quantity must be infiltrated undiluted in the lesions. We can only speculate whether the use of HRIG, or indeed refraining from suturing the wounds, could have prevented the course of disease in this case. The second case concerned a boy who only received treatment on the day he was bitten by a rabid dog, although this was not confirmed. With no subsequent vaccinations the boy died. What is notable in this case is that a cousin bitten by the same animal on the same day, and who completed the recommended rabies vaccination series through 90 days, was in good health 10 months post exposure. These two cases are defined as rabies PEP intervention failures.

In summary, records of 7,660 patients given rabies PEP at the RITM during the period July 2003 to August 2004 were considered in this case series. Although the true extent of rabies exposure in all 7,660 patients is unknown, of 144 cases of laboratory-proven rabies Category III exposure available for follow-up, there was one rabies PEP intervention failure. A second presumed PEP intervention failure, there being no confirmation of rabies infection in the biting animal, highlights the importance of the potential of rabies infection by animals not being examined for their rabies status and illustrates that the burden of disease is more important.

Thus, the RITM rabies PEP guidelines, *i.e.*, wound cleaning and treatment, antibiotic and tetanus prophylaxis, rabies vaccination and use of pERIG for Category III exposures by potential rabid animals are deemed satisfactory. Whereas further consideration of the development of alternative treatments to the current RIGs, such as monoclonal antibodies, is merited [11], the real-world, clinical experience presented here emphasizes that, in the meantime, correct implementation of the rabies prevention recommendations is of paramount importance to save lives.

Continued training of treating physicians and attending staff should further improve the quality of treatment and care. Extensive lesions in highly innervated body regions are of particular concern and may demand specific clinical interventions, such as immediate suturing. It must be borne in mind that such deviations from the recommendations are often basic and unavoidable when working under field conditions, and must be considered with great caution when interpreting unexpected outcomes. The experience from the RITM is that pERIG, when administered as recommended and as part of the rabies PEP in conjunction with wound treatment and rabies vaccination, is safe and effective and contributes to the prevention of otherwise fatal consequences of rabies infection.

## References

[1] Rupprecht CE (2004). A tale of two worlds: public health management decisions in human rabies prevention (editorial commentary).. Clin Infect Dis.

[2] Anonymous (2005). WHO Expert Consultation on Rabies: First Report. Technical Report Series 931.. http://www.who.int/rabies.

[3] Servat A, Lutsch C, Delore V, Lang J, Veitch K, Cliquet F (2003). Efficacy of rabies immunoglobulins in an experimental post-exposure prohylaxis rodent model.. Vaccine.

[4] Lang J, Attanah P, Quiambao B (1998). Evaluation of the safety, immunogenicity, and pharmacokinetic profile of a new, highly purified, heat-treated equine rabies immunoglobulin, administered either alone or in association with a purified, Vero-cell rabies vaccine.. Acta Tropica.

[5] Robles CG, Miranda NLJ (1992). Comparative evaluation of the rabies fluorescent antibody test and direct microscopic examination at the Research Institute for Tropical Medicine.. Phil J Microbiol Infect Dis.

[6] Anonymous (1996). Recommendations on rabies post-exposure treatment and the correct technique of intradermal immunization against rabies..

[7] Ali NS (2003). Guidelines for prophylaxis of rabies in Pakistan.. Pak J Med Sci.

[8] Chantanakajornfung A, Narapron N, Khumphai W, Bejavongkulchai M, Mitmoonpitak C, Wilde H (1999). A study of human rabies immune globulin manufactured by the Thai Red Cross.. Vaccine.

[9] Sudarshan MK, Madhusudana SN, Mahendra BJ (2007). Assessing the burden of human rabies in India: results of a national multi-center epidemiological survey.. Int J Infect Dis.

[10] Suwansrinon K, Jaijaroensup W, Wilde HY, Sitprija V (2006). Short Report: is injecting a finger with rabies immunoglobulin dangerous?. Am J Trop Med Hyg.

[11] Hanlon CA, Niezgoda M, Morrill PA, Rupprecht CE (2001). The incurable wound revisited: progress in human rabies prevention?. Vaccine.

[12] Quiambao BP, Laot T, Castillo Y, Teuwen DE, Zinsou J-A (2005). Rabies post-exposure prophylaxis (PEP) policy at RITM, Manila, Philippines (*abstract*) XVIth International Conference on Rabies in the Americas, Ottawa, Canada..

[13] Sampath G, Parikh S, Sangram P, Briggs DJ (2005). Rabies post-exposure prophylaxis in malnourished children exposed to suspect rabid animals.. Vaccine.

